# *Halophila beccarii* extract ameliorate glucose uptake in 3T3-L1 adipocyte cells and improves glucose homeostasis in streptozotocin-induced diabetic rats^☆^

**DOI:** 10.1016/j.heliyon.2022.e10252

**Published:** 2022-08-15

**Authors:** Vani Mathakala, Muni Kesavulu Muppuru, Uma Maheswari Devi Palempalli

**Affiliations:** a Department of Applied Microbiology and Biochemistry, Sri Padmavati Mahila Visva vidyalayam, Tirupati, AP, India; b Department of Biosciences, Mohanbabu University, Sree Vidyanikethan Engineering college, Sree Sainath Nagar, Tirupati, AP, India

**Keywords:** HBE, Glucose uptake activity, Molecular docking, T2DM, GLUT4, Metabolic enzymes

## Abstract

The regulation of carbohydrate metabolizing enzymes is an effective way of reducing blood glucose levels and improving glycogen synthesis during the management of type 2 diabetes. The present investigation was conducted to explain the detailed mechanism with which a Seagrass, *Halophila beccarii* extract (HBE) enhances the glucose uptake in the 3T3-L1 adipocyte cell culture system in invitro. HBE stimulates the glucose uptake by the translocation of glucose transporter 4 (GLUT4) on to plasma cell membrane through induction of insulin receptor substrate 1 (IRS-1)/protein kinase B (Akt) signaling pathways. To assess the effect of HBE on T2DM, we used invivo experimental diabetes rat models induced with streptozotocin (STZ) to perform oral GTT and ITT. Furthermore, we assessed the enzymatic profile of Glycolysis, Pentose phosphate pathway, and gluconeogenesis from liver tissue homogenate. After long-term exposure with HBE, our results confirmed, that HBE improves the glucose uptake in 3T3-L1 cell lines by up-regulation of glucose transporter type 4 (GLUT4) through uptake of glucose by the adipocytes. The resulting data indicated that HBE had a great potentiality in preventing diabetes and maintaining glucose homeostasis through improving glucose uptake. The present data also showed that HBE with its insulin mimetic activity activates glycogen synthesis and enhances glucose utilization by regulating the carbohydrate metabolic enzymes. The similarity between HBE and insulin indicates that the HBE follows the mechanisms same as the insulin signaling pathway to show the antidiabetic activity.

## 1 Introduction

Diabetes Mellitus, a lifestyle metabolic disorder of complex aetiologies exemplified with failing of enzymes involved in carbohydrate, fatty acid, and protein metabolism. The disturbances in metabolic pathways are attributed to defects in insulin secretion due to the dysfunction of β-cells or insulin resistance or a combination of insulin resistance and β-cell dysfunction [1]. The defective secretion of β-cells, metabolic turbulence, and pathological manifestations in tissues like the pancreas, eyes, liver, muscle, adipose tissue, kidney, and nerves contributes to the perturbations in the physiological glucose concentration [2]. The root cause for hyperglycemia in diabetes mellitus is overproduction of Glucose attributed to increased glycogenolysis and gluconeogenesis and also decreased utilization of glucose by tissues [3, 4]. Glucose homeostasis is maintained in the body due to the balance between catabolism and anabolism of glucose after its absorption from the intestine. The catabolic route of glucose through glycolysis, pentose phosphate pathway, the citric acid cycle is in equilibrium with anabolism of glucose through glycogen synthesis, gluconeogenesis, and glycogenolysis [5]. Glucose homeostasis prevails through the continuous supply of glucose to the cells. Failure in the regulation of hepatic glucose synthesis in the absorptive state and excess production of glucose in the post-absorptive state are the leading cause of hyperglycemia in diabetes [6]. Thus, the regulation of key hepatic enzymes is the potential target for the maintenance of glucose production in liver cells and thereby controls the blood glucose levels in diabetes. Assay of Glucose uptake activity in the 3T3-L1 adipocytes cell line through the GLUT4 translocation on the plasma membrane is one of the significant target mechanisms to attain glucose homeostasis and is considered to be an efficient target for the development of proficient drugs for diabetes and obesity [7, 8, 9, 10]. However, the detailed mechanism of this anti-diabetic activity has not yet been understood. Therefore, there is a need for screening natural products with anti-hypoglycemic activity targeting glucose metabolism.

Currently, much of the research is focused on marine sources like seagrass, algae, actinomycetes for characterizing the suitable hypoglycemic agents [11]. Seagrasses are submerged marine plants located in tidal and sub-tidal marine environments. The majority of the seagrass plants belonging to the family Hydrocharitaceae are found on the southeast coast of Tamil Nadu, Andaman, and Nicobar islands. Seagrass meadows closely resemble the ecosystem of mangroves and coral reefs.

Our study had demonstrated that oral administration of Seagrass, *Halophila beccarii* extract (HBE) at a dose of 500 mg/kg bwt enhances the production and translocation of GLUT4 in adipocytes tissue cell line [12]. In this present evaluation, we probed the elaborated molecular basis employing the anti-diabetic effect of HBE mainly focusing on the insulin signal pathways which mediated glucose uptake in 3T3-L1 adipocytes. To investigate the effect of HBE on type 2 diabetes in vivo, we further analyzed the hepatic key enzymes which play a key role in glucose utilization in non-diabetes conditions and oral glucose tolerance tests and insulin tolerance tests in type 2 diabetes model rats administered with HBE through oral gavage. The HBE extract was screened for antioxidant activity and screening of active compounds through GC-MS analysis [13]. Additionally, molecular docking simulation studies were performed to predict the docking scores of ligand in each targeted protein involved.

## 2 Materials and methods

### 2.1 Preparation of HBE

The seagrass plants were collected from the sea coast of Bay of Bengal, A.P, India and reported as *Halophila beccarii*. The extract was prepared by dissolving 20 g of dried powder of *H. beccarii* in 200 ml of methanol forseven daysatroom temperature. The solution was then filtered using a Whatman No. 1 filter paper, and the extract was evaporated to dryness with a Rota evaporator under reduced pressure at 40 °C. The dried extract wasdissolved in 0.1% DMSO and stored at 4 °C for further use. and the extract was designated as HBE [14].

### 2.2 Gas chromatography–mass spectrometry (GC-MS) analysis

Gas Chromatogram-mass spectral analysis (GC-MS) was carried out to map the bioactive compounds of seagrass *H. beccarii*. The dried methanolic (95% v/v) extract of seagrass was loaded onto GCMSQP02010 (SHIMADZU)and equipped with Turbo mass gold-pekin Elmer Detector and split injection system. Throughout the experiment analysis, temperature was maintained at 250 °C. Electron energy level of 70 ev was used to analyze the mass spectra of the extract between 45 m/z and 450 m/z for about 45 min duration.

### 2.3 Cell culture

3T3-L1 fibroblast cell lines obtained from the National center for cell sciences were cultured and differentiated into adipocytes by the standard method [15]. 3T3-L1adipocytes were cultured in DMEM in a 5% CO2 incubator for about 72 h. After 75% of confluence, the DMEM medium was replaced with a differentiation medium and maintained for about 8 days to convert preadipocytes to mature adipocytes. The mature adipocytes were detected based on the lipid droplets after fixation with formaldehyde and stained with 0.5% Oil-Red O stain [16].

### 2.4 Bio compatibility of HBE on 3T3 L1 adipocyte cell line

MTT assay (3-(4.5-dimethylthiazole-2-yl)-2.5-diphenyltetrazolium bromide) was adopted to analyze the Cytotoxicity of HBE [17, 18] on 3T3 L1 adipocyte cell lines based on mitochondrial respiration. The percentage cell viability was detected based on the formazan crystals formed by mitochondrial succinate dehydrogenase. Cytotoxicity was expressed as a percentage of cell viability considering 100% viability in the solvent control (0.5% DMSO).

### 2.5 Glucose uptake assay

The impact of HBE on uptake of glucose was measured by using 3T3 L1 adipocytes cell lines. The differentiated cells were serum-starved overnight and during the experimental analysis, the cells were washed with HEPES Krebs ringer phosphate buffer (KRP) and incubated with 0.1% BSA for about 30 min at 37 °C. After incubation, the cells were treated with glibenclamide, insulin, different concentrations of HBE (100–1000 μg/ml) independently for 30 min, and negative control was maintained. After 30 min, the cells were treated with D-glucose at 37 °C for 30 min. The uptake of glucose was terminated by the removal of glucose solution from wells and washed with ice-cold KRP buffer. Further, the cells were lysed with 0.1 M NaOH and an aliquot of cell lysate was used to measure cell-associated glucose [[19], [20]]. Theglucose assay kit was purchased fromSwamy distributors, Tirupati, AP, IndiaLinear detection range in 96-well plate: 5–300 μM (90 μg/dL to 5.4 mg/dL) glucose.

### 2.6 Experimental animals

Male albino rats (180–200 g) were maintained on a standard pellet diet and provided access to water and libitum. They were housed in a cage in a temperature-controlled room at 22–23 °C with a 12 h photoperiod. Animals were divided into five groups Normal control (NC), Normal treated with HBE (500 mg/kg bwt) (NT), STZ-induced diabetic control (DC), Diabetic rats treated with HBE (STZ + HBE 500 mg/kg bwt), (DT)Diabetic rats treated with glibenclamide (DG). The HBE and standard drug glibenclamide was supplemented to all the experimental rats at regular intervals of 24 h by oral gavage for about 40 days. All the experiments were carried out under the approval of an Animal Ethical Committee of Sri Padmavati Mahila Visvavidyalayam, Tirupati (1677/Po/S/2012/CPCSEA/30)**.**

### 2.7 Oral glucose tolerance (OGT) and insulin tolerance (IT)Test

OGT and IT tests were performed both in control and diabetic rats treated with HBE and standard glibenclamide. All the rats of each group except the control were orally ingested with HBE (500 mg/kg bwt) for 40 days. A glucose tolerance test was performed by fasting the rats for six hours followed by oral administration of glucose 5 g/kg bwt. Blood glucose levels were estimated by glucometer using a blood collected from a tail vein puncture. Insulin tolerance test was done in 4 h fasted animals administered with insulin intraperitoneally at a dose level of 0.75 U/kg bwt followed by glucose estimation. The insulin profile in the plasma of experimental animals was quantified with ELISA as per the standard protocol (Boerhringer Mannheim kit).

### 2.8 Biochemical measurements

To measure the impact of HBE on glycolytic enzymes, 250 mg of the chilled liver of the treated rats was homogenized at 4 °C with 0.1 M citrate buffer at pH 6 (1:1 w/v) and centrifuged at 3000 rpm for 10 min). The assay of Hexose kinase was carried out by the addition of 2.5 ml of reaction mixture (1 ml of glucose, 0.5 ml of ATP, 0.1 ml of MgCl_2_, 0.1 ml of NaF, 0.4 ml of KH2PO4 and 0.4 ml of KCl) to 0.2 ml of tissue homogenate and the amount of glucose in the supernatant was estimated by the O-toluidine method [21], Gluconeogenic enzymes such as glucose-6-phosphatase and fructose-1,6-bis phosphatase were asssed as per the standard mehods. In brief, the liver tissue homogenate was treated with 0.3 ml of citrate buffer and 0.5 ml of glucose 6- phosphate or fructose-1,6-bis phosphateand the reaction was terminated with 1 ml 10% after 1h incubation at 37 °C.Further, the released inorganic phosphate was estimated by the standard methods [22, 23] The activity of Glucose-6- phosphate dehydrogenase of pentose phosphate pathway was calculated based on the rate of oxidation-reduction of Glucose-6-phosphate and NADP respectively. The reaction initiated with the addition of 0.1 ml of tissue extract to the assay mixture containing 2.5 ml of buffer, 0.1 ml of glucose-6-phosphate, 0.2 ml of NADP+, 0.1 ml of MgCl2. The activity of the enzyme was determined by using molar extinction coefficient of NADPH at 6.22cm1mM-1 [24].

### 2.9 Molecular docking of GLUT4 transporter protein

To predict full length structures of human glucose transporter type (GLUT) 4, sequence was retrieved from UniProt web site (http://www.uniprot.org/; UniProt Entry ID: P11168 and P14672) and subjected to BLASTp program to obtain their homologue structures from Protein Data Bank. After BLASTp, the obtained PDB IDs 5EQG, 4PYP and 4ZW9 were selected as templates for modeling of GLUT 4 structure. The selected templates 5EQG and 4PYP shares 65% identity and 97% query coverage, whereas 4ZW9 shares 65% identity and 94% query coverage. Further, the aligned sequences were used to build model structures using MODELERE program (https://salilab.org/modeller/). Total 10 models were generated for GLUT4 and the structure was validated with PROCHECK and ProSa web servers and the best reliable structure of GLUT4 was chosen for further study (12). The Phytochemicals of HBE reported through GC analysis (data not shown) such as Hexadecanoic acid (CID:985), Cis-9 Octadecenoic acid (CID:445639), Methyl palmitate (CID:8181), Beta-sitosterol (CID: 222284), isocoumarin (CID:68108), 1,2 benzene dicarboxylic acid (CID:1017) and reference compounds (metformin-4091, glibenclamide-3488) were selected as ligands and sketched using ‘Prepare Ligand’ module of DS. AutoDock program was used to study the binding of the ligand within the active site of GLUT4 transporter protein and binding characters were envisioned using the Molecular display program PyMOL [25].

### 2.10 Statistical analysis

Analysis of variance (ANOVA) and Duncan's multiple comparison test (DMCT) was performed to calculate the statistical significance of the experimental data. Statistical significance is denoted by an asterisk (∗) when p values are ∗p < 0.03 to 0.05, ∗∗p < 0.001, ∗∗∗p < 0.0001 and ∗∗∗∗p < 0.00001.

## 3 Results

In the current study, the whole plant of *Halophila beccarii* was extracted with methanol.

### 3.1 Differentiation, cell viability and glucose uptake activity of adipocytes

**Figure 1:**
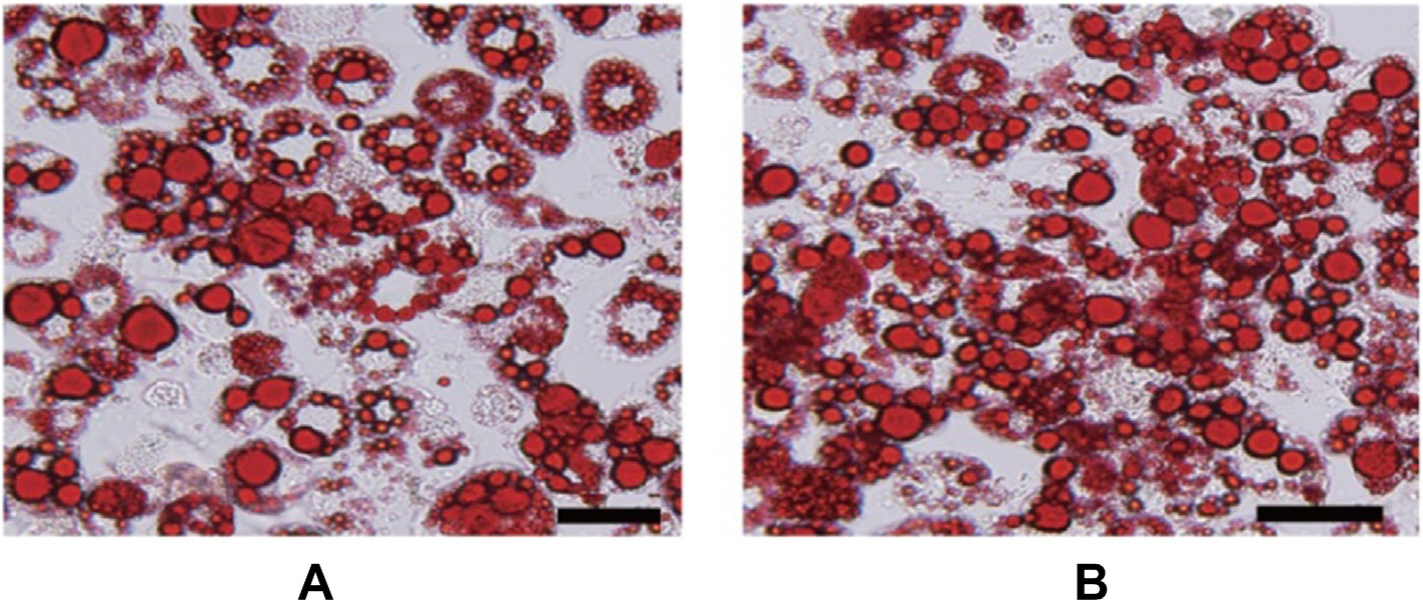
In situ image of Adipocytes differentiated from mouse 3T3-L1 fibroblasts (A) HBE treated Oil red O stain of adipocyte cells and (B) Oil red O stain differentiated 3T3-L1 cells. Bars: A. 100 μm, B. 50 μm.

The intracellular lipid accumulation, the hallmark of mature adipocytes, was detected through Oil-Red O staining in 3T3-L1 cell lines). Figure 1A showed the correlation of Oil-Red O staining with lipid accumulation in differentiated 3T3- L1 cells. The Cytotoxicity of HBE against 3T3L1 adipocytes was assessed through MTT assay which has been a widely accepted method to assess the cell viability based on mitochondrial respiration. HBE was found to be nontoxic and the percentage proliferation of HBE treated cells was on par with the control cells of 3T3 L1 adipocytes even at 500 μg/ml (Table 1) which confirms the cytoprotective nature of HBE [26, 27]. The glucose uptake activity of HBE was compared with insulin (1 U/ml) and glibenclamide (100 μg/ml). The HBE at a dose of 500 μg/ml represented the equal percentage of glucose uptake with glibenclamide (100 μg/ml) and at 1000 μg/ml the uptake activity of HBE is nearly equal with insulin (Table 2).

**Table 1:** Proliferation of 3T3 L1 adipocyte cell line in the presence of HBE extract.

S. No	Concentration of HBEμg/ml	% of Viability
1.	Control	100
2.	100	98.1 ± 1.12
3.	200	97.5 ± 1.07
4.	300	96.5 ± 1.91
5.	400	95.8 ± 0.25
6.	500	94.2 ± 1.06

**Table 2:** Percentage of glucose uptake after treatment with various concentrations HBE on 3T3-L1 adipocyte cell.

S. No	Concentration of HBE	% of Glucose uptake
1.	100	40 ± 1.21
2.	500	65 ± 2.07
3.	750	78 ± 2.51
4.	1000	85 ± 0.25
5.	Insulin I U/ml	92 ± 1.06
6	Glibenclamide	65 ± 0.93

### 3.2 Effect of HBE on OGT(Oral glucose tolerance test) and ITT (insulin tolerance test)

**Figure 2:**
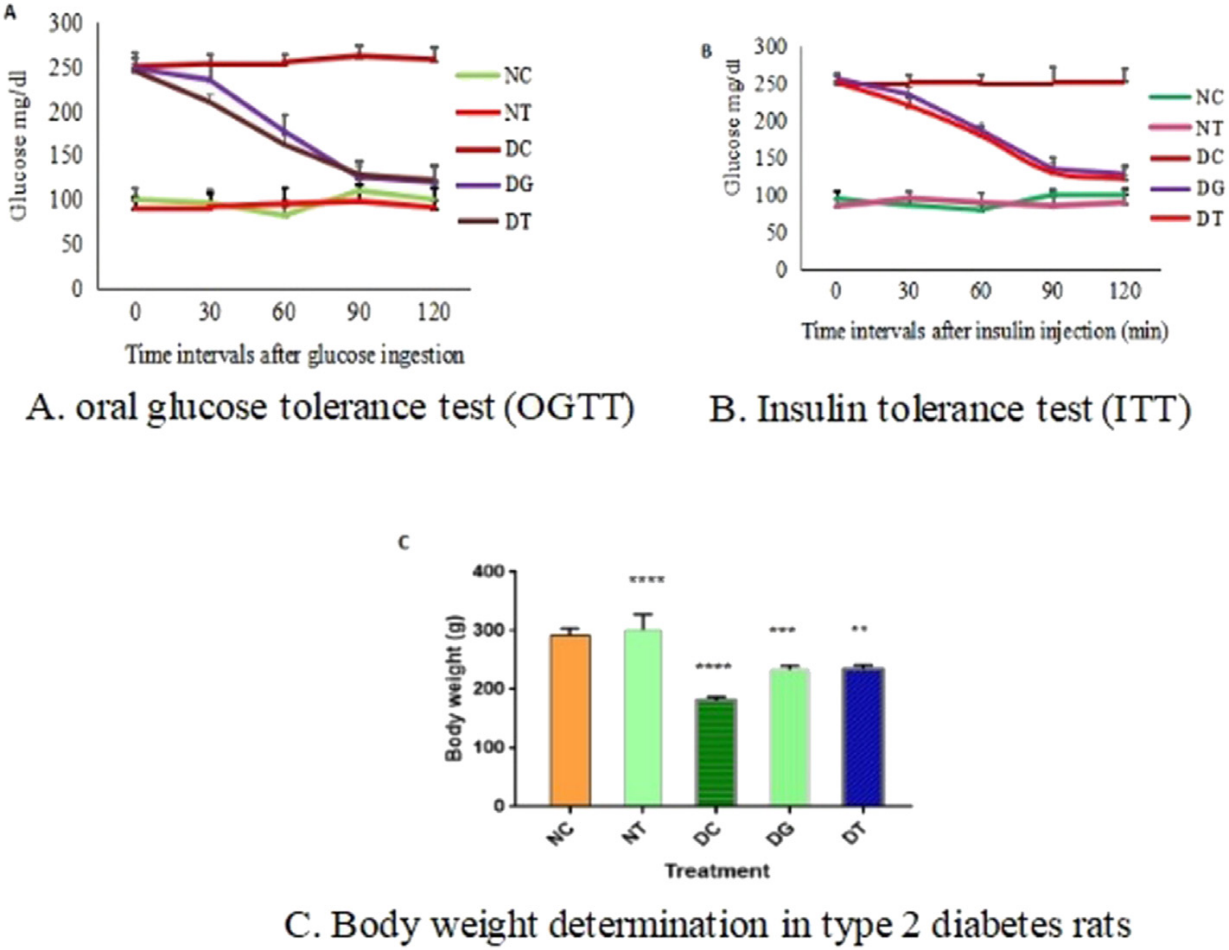
Effect of HBE administration on OGT and insulin intolerance in type 2 diabetic rats. A. For oral glucose tolerance test (OGTT) B. For insulin tolerance test (ITT). C. Body weight determination in type 2 diabetes rats. Statistical significance is denoted by an asterisk (∗) when p values are ∗p < 0.03 to 0.05, ∗∗p < 0.001, ∗∗∗p < 0.0001 and ∗∗∗∗p < 0.00001.

OGTT (Oral glucose tolerance test) and ITT (Insulin Tolerance test) were performed by using STZ-induced diabetes model rats (Figure 2(A–C)). In OGTT, HBE treated diabetes rats represented improved glucose tolerance levels compared with disease control and HBE didn't show any glucose-lowering capacity and body weight in normal treated rats. Similarly, HBE treatment initiated the regulation of glucose by enhancing insulin tolerance capacity in STZ diabetes rats after 120 min of injection with insulin.

### 3.3 Molecular docking

**Figure 3:**
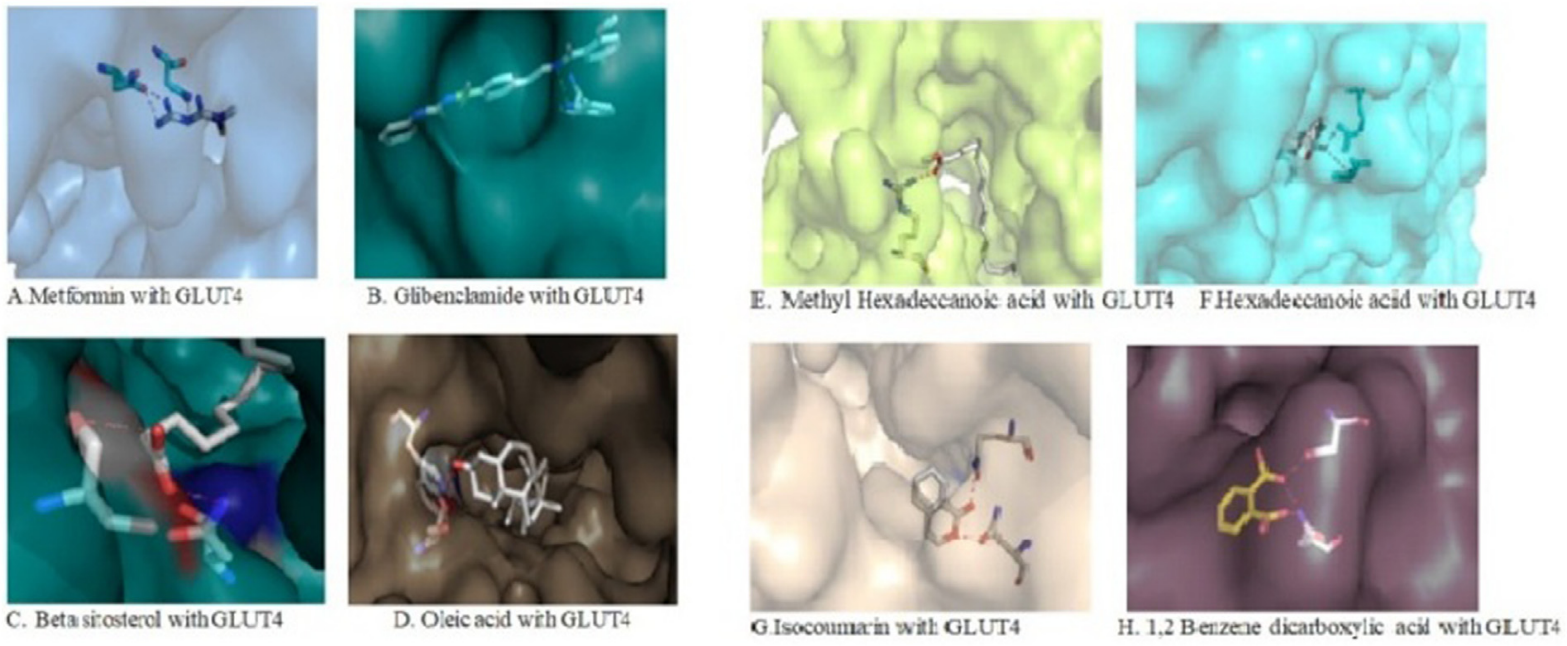
A. Active site in GLUT4 transporter protein with (A) Metformin (centre blue) interact with GLUT4 at active site (B) Glibenclamide (centre green) interact with GLUT4 at active site (C) Betasitosterol (centre white) interact with GLUT4 (brown) At active site (D) Cis-9 Octadecanoic acid (centre red) interact with GLUT4 (Green) at active site Figure 3B. (E) Methyl hexadecanoic acid (centre) interact with GLUT4 (Green) at active site(F) Hexadecanoic acid (cetre) interact with GLUT4 (blue) at active site (G) Isocoumarin acid (centre red) interact with GLUT4 (brown) At active site(H) 1,2 Benzene dicarboxylic acid (centre green) interact with GLUT4 (brown) At active site.

Molecular docking was performed to analyze the binding interaction of selected compounds with active pocket sites of GLUT4 transporter. The more negative value indicates the better binding affinity with the target protein (Iman M, 2015). Among all the compounds docked, Beta-sitosterol (−49.86), Cis-9 Octadecanoic acid (−36.94), Hexadecanoic acid (−34.42), Methyl palmitate (−33.71) showed stronger binding energy than isocoumarin (−22.25) and 1,2 benzene dicarboxylic acid (−13.28), and the binding energies were found to be greater compared with reference compounds metformin (−16.41), glibenclamide (−54.78) (Figure 3A and B). The most possible chemical attraction and H-bond interaction details of the ligands at targeted protein active sites, and their corresponding energy values were listed in Table 3, respectively.

**Table 3:** Docking energy values and interactions of seagrass bioactive metabolites and standard antidiabetic with GLUT4 protein.

Compound name	Binding energy (kacl/mol)	Amino acids involved in interaction	Ligand atom involved in interaction	H-Bond distance	Attractive van der Waals energy	Repulsive van der Waals energy	Atomic contact energy
Metformin	−5.73	Asn 427	N1	2.21	−5.73	0.33	−5.61
		Asn 431	N2	2.71			
			N3	2.40			
Glibenclamide	−21.62	Trp 428	N3	2.59	−21.62	3.38	−16.52
		Ser 153	O4	3.13			
Beta sitosterol	−21.72	Trp 428	O	2.48	−21.72	8.31	−14.99
		Ser 96	O	3.05			
Cis-9 Oleic acid	−17.10	Asn 176	O2	2.41	−17.10	3.69	−9.04
		Ser 153	O1	2.89			
Hexadecanoic acid	−13.87	Thr 337	O1	3.15	−13.87	2.63	−10.23
			O1				
Methyl HDA	−16.41	Arg 416	O2	2.40	−16.41	8.51	−10.65
Isocoumarin	−7.84	Asn 304	O2	2.90	−7.84	0.35	−7.49
		Gln 299	O2	2.49			
1,2 Benezene di carboxylic acid	−9.94	Asn 431	O2&O3	2.10&2.28	−9.94	1.85	−0.63
		Asn 304	O3	2.99			

### 3.4 Regulation of glucose metabolic and gluconeogenesis enzymes by HBE in STZ induced diabetic rats

The nontoxic nature of HBE extract was noticed at doses up to 500 mg/kg bwt. All the animals were alive, healthy, and lethal or toxic reactions were not found during 40 days of the long-term experimental period.

**Figure 4:**
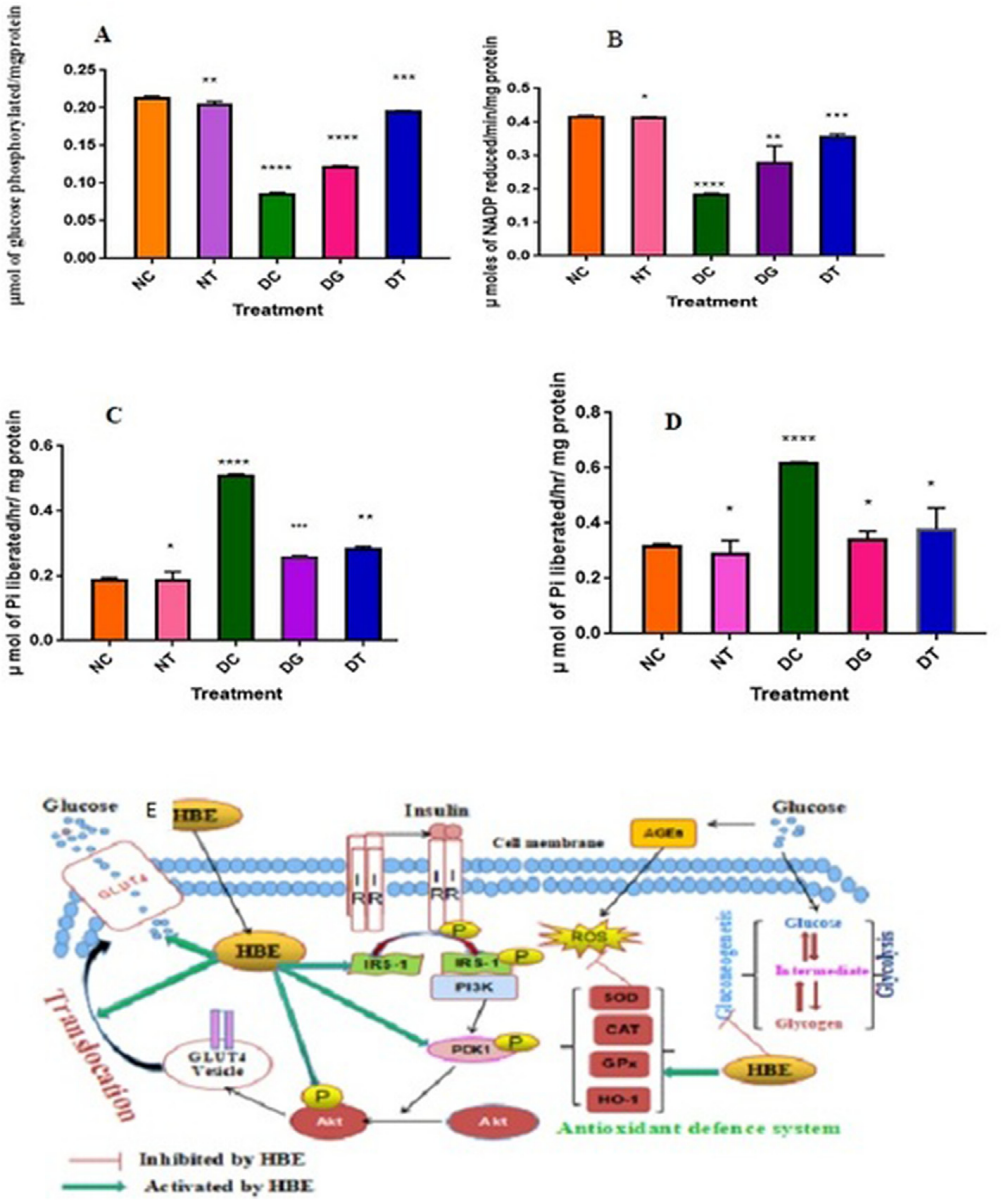
A. Effect of HBE on hepatic Hexokinase in normal and diabetic rats **B.** Effect of HBE on hepatic Glucose-6-phosphate dehydrogenase in STZ-diabetic rats. **C.** Effect of HBE on hepatic Glucose-6-phosphatase and **D.** Fructose 1,6- bisphosphatase in normal and diabetic rats.**E. P**roposed mechanism of action of *Halophila beccarii* extract (HBE) in glucose homeostasis and GLUT4 translocation through IRS-1/Akt signaling cascade activation and gluconeogenesis repression. Statistical significance is denoted by an asterisk (∗) when p values are ∗p < 0.03 to 0.05, ∗∗p < 0.001, ∗∗∗p < 0.0001 and ∗∗∗∗p < 0.00001.

The diabetic control showed the inhibition of glucose degradation by glycolysis due to decreased activity of Hexokinase. Both Glibenclamide and HBE treated animals showed a significant increase in Hexokinase activity (Figure 4A) which demonstrates the utilization of glucose in the liver. Hexokinase levels were statistically significant and the phosphorylation seems to be high with HBE than glibenclamide. As shown in Figure 4B, the STZ-diabetic control group showed 50% inhibition of NADP reduction due to decreased activity of Glucose-6-Phosphate dehydrogenase. After 40 days of oral administration, the HBE stimulated the activity of glucose-6-phosphate dehydrogenase by resuming the oxidation of Glucose 6-phosphate and reduction of NADP. The hepatocytes were selected to assess the impact of HBE on Glucose-6-Phosphatase which is one of the key enzymes in the synthesis of glucose from non-carbohydrate precursors. The release of inorganic phosphate, an indicator for Glucose-6-Phosphatase activity, was found to be very high in STZ-diabetic rats compared to normal control rats. The HBE treatment reduced the release of inorganic phosphate by limiting the activity of Glucose-6-phosphatase (Figure 4C). The reduced activity of Glucose-6-phosphatase by HBE indicates the effective operation of the pentose phosphate pathway. Induction with STZ stimulated the activity of Fructose 1, 6 bis-phosphatase to 2.5 folds higher compared to a normal group (Figure 4D). The treatment with HBE and Glibenclamide reduced the release of inorganic phosphate by limiting the activity of Fructose 1, 6 bis-phosphatase activity.

## 4 Discussion

Diabetes mellitus is a puzzling metabolic disease with serious unidentified health implications. Several drugs individually or in different combinations such as Insulin, insulin secretagogues, insulin sensitizers, and prandial glucose regulators are available to accomplish better glycemic control [28]. In the current study, we revealed that HBE significantly improves glucose homeostasis by regulating gluconeogenesis and glycolysis through enzymatic analysis. The experimental work portrayed the effect of seagrass *Halophila beccarii* on metabolic enzymes of glucose in the liver of STZ-diabetic rats [3]. Different doses of HBE (250, 500, and 750 mg/kg bwt) were tested for anti-hyperglycemic activity in a short-term study. The HBE showed an anti-hyperglycemic effect by decreasing the fasting blood glucose levels up to 52% in STZ-diabetic animals within 6 h [12]. The HBE extract at a dose level of 500 mg/kg bwt exhibited pronounced antidiabetic activity. Treatment with HBE and glibenclamide for 40 days, demonstrated normal levels of blood glucose levels in STZ-diabetic rats. The data confirms the anti-hyperglycemic activity of HBE instead of hypoglycemic activity is due to the presence of a significant amount of phytochemicals such as phenols and flavonoids in the extract [14]. The feasible mechanism of antihyperglycemic activity of HBE might be due to bio-active fatty acids and phenols of *Halophila beccarii* which play an important role in the regeneration of pancreatic β cells [29]. The changes were well supported with the regulated levels of carbohydrate metabolizing enzymes. These phytoconstituents might perform insulin-like functions either by uptake of glucose or inhibition of hepatic gluconeogenesis [30].

Hepatic gluconeogenesis is the main contributor to postprandial and fasting hyperglycemia in diabetes and is also required to achieve glucose homeostasis [31]. The uncontrolled activity of gluconeogenic enzymes in the cell leads to increases in the fasting blood glucose levels in diabetes. In normal individuals, Insulin can balance gluconeogenesis through enhanced glycogenesis and glycolysis mechanisms. In diabetes, the gluconeogenesis enzymes are over-expressed and glycolytic enzymes are decreased in their level of activity. Biologically there is one such mechanism to inhibit the gluconeogenesis pathways, such as either inhibition of key genes and glucose 6 phosphatase genes transcriptional activators [32] or inhibition of enzyme activity. In the current study, HBE decreases the expression of glucose 6-phosphatase and fructose 1, 6- bis phosphatases enzymes in the liver of STZ diabetes control group rats. The data propose the role of HBE in the translocation of GLUT4 proved by the up-regulation of glycolytic enzymes by repressing the gluconeogenic pathway.

HBE treatment attains the phosphorylation of insulin signaling cascade proteins, one of the phosphorylated Akt boosted GLUT4 expression and attenuated the gluconeogenesis pathway by downregulating the glucose-6- phosphatase (G6Pase) and fructose 1,6- bis phosphatase in the liver and reciprocally improve the glucose metabolism through glycolysis pathway (Figure 4E). In our findings, HBE enhances the GLUT4 stimulation and may improve the mechanism of hypoglycemia and antidiabetic drugs.

To study the glucose uptake and GLUT4 translocation in a cellular model, the 3T3-L1 cell lines are the best characterized model. The action in the uptake of glucose in adipose tissue is the primary target site in glucose metabolism and to obtain glucose homeostasis by controlling hyperglycemia through insulin-stimulated glucose uptake [33]. 3T3-L1cells are having an intact insulin signaling pathway to express the insulin-sensitive GLUT4 [34]. Based on the findings, HBE showed increased glucose uptake with increased concentrations. This is in agreement with the previous studies done in medicinal plants [35]. Adipocytes are used as in-vitro models to confirm the translocation of GLUT4 through uptake of glucose by the 3T3 L1 cells. As per the data mentioned in Fig-1C, the glucose uptake activity of adipocytes was compared with glibenclamide. Which demonstrates the translocation of GLUT4. Further, docking analysis showed the activation of GLUT4 protein by the phytochemical constituents of HBE which is an indicator for the translocation of GLUT4. The presence of bioactive compounds in HBE leads to promote glucose absorption and can act as a synergistic interaction with multiple targets to enhance a pharmacological action in T2DM maintenance.

In docking results, the lowest binding energy value represents the active protein-ligand interaction stability. When compared with standard antidiabetic drugs metformin and glibenclamide docking result with HBE bioactive compounds, 1,2 benzene dicarboxylic acid, isocoumarin, Cis-9 octadecenoic acid, and methyl hexadecanoic acid significantly had almost near binding value and highest hydrogen bong length with GLUT4. With these binding affinities, the HBE shows a more potent stimulatory effect on GLUT4 vesicles [36]. The binding poses of the extract with GLUT4 demonstrate the active site pocket of GLUT4 protein, which explains the stimulatory of HBE on GLUT4 translocation and glucose uptake at a cellular level and thus brought back to the normal glycemic levels in type 2 DM. The HBE extended hydrogen interactions with amino acids such as Asn 427, Trp 428, Ser 96, Trp 428, ser 96, Ser 153, Thr 337, Arg 416, Gln 299, and Asn 304 of Glucose transporter-4. The site of action of Cis -9 Oleic acid 1,2 benzene dicarboxylic acid, isocoumarin, and methyl hexadecanoic acid of HBE was found to be Trp 428 of GLUT4 which is correlated with Glibenclamide and metformin. In-silico analysis demonstrated that Asn 427, Trp 428, Ser 153, Ser 96, and Gln-299 residues are catalytically significant to comprehend the molecular activity of GLUT4 protein.

In conclusion, The present study inferred that HBE strongly inhibits gluconeogenesis activity in STZ induced diabetes rats. HBE also speeds up glucose transport in 3T3-L1 adipocytes cell lines by inducing the translocation of GLUT4 via an insulin-linked signaling pathway. In an in vivo study, HBE significantly revised oral glucose tolerance and insulin tolerance in the STZ-induced diabetic rat model. The effect of HBE compounds that exhibited antihyperglycemic activity was also studied exhaustively by molecular docking that revealed all the screened compounds act as fortunate candidates which dock significantly with targeted protein GLUT4 and make it possible to the expression of GLUT4 effectively in the absorption and metabolism of glucose. Instead of a single isolated compound, the methanolic extract of *Halophila beccarii* itself acts as a prominent drug to reduce hyperglycemia without side effects as an insulin-sensitizer.

## Declarations

### Author contribution

Vani Mathakala: Performed the experiments; Analyzed and interpreted the data.

Muni Kesavulu Muppuru: Analyzed and interpreted the data.

Uma Maheswari Devi Palempalli: Conceived and designed the experiments; Contributed reagents, materials, analysis tools or data; Wrote the paper.

### Funding

This research did not receive any specific grant from funding agencies in the public, commercial, or not-for-profit sectors.

### Data availability

Data included in article/supp. material/referenced in article.

### Declaration of interest

The authors declare no conflict of interest.

### Additional information

No additional information is available for this paper.
